# Computed tomography and magnetic resonance imaging study of a normal tarsal joint in a Bengal tiger (Panthera tigris)

**DOI:** 10.1186/s12917-019-1865-1

**Published:** 2019-04-29

**Authors:** Alberto Arencibia, Jorge Matos, Mario Encinoso, Francisco Gil, Alejandro Artiles, Francisco Martínez-Gomariz, José Maria Vázquez

**Affiliations:** 1 Department of Morphology, Veterinary Faculty, University of Las Palmas de Gran Canaria, Trasmontaña s/n, 35413 Arucas, Gran Canaria Spain; 2 Veterinary Hospital Los Tarahales, Recta de Los Tarahales 15, 35013 Las Palmas de Gran Canaria, Spain; 3 Department of Anatomy and Comparative Anatomy, Veterinary Faculty, University of Murcia, Campus de Espinardo, 30071 Murcia, Spain

**Keywords:** Computed tomography, Magnetic resonance imaging, Anatomy, Tarsal joint, Tiger

## Abstract

**Background:**

In this research, using computed tomography (CT) and magnetic resonance imaging (MRI), we provide a thorough description of the standard appearance of a right tarsal joint in a Bengal tiger (*Panthera tigris*). CT scans were performed using a bone and soft tissue window setting, and three-dimensional surface reconstructed CT images were obtained. The MRI protocol was based on the use of Spin-echo (SE) T1-weighted and Gradient-echo (GE) STIR T2-weighted pulse sequences. Magnetic resonance (MR) images were taken in the transverse, sagittal and dorsal planes. We also performed anatomical dissections to facilitate the interpretation of the different structures of the tarsus joint and allow comparisons with CT and MRI images.

**Results:**

The CT images allowed us to observe differences between the bones and soft tissues of the tarsal joint. When applying the bone window setting, the obtained footage showed the anatomy between the medulla and cortex. Additionally, the trabecular bone was delineated. By contrast, the soft tissue window allowed the main soft tissue structures of the tarsal joint, including ligaments, muscles and tendons, to be differentiated. Footage of the main anatomical structures of the standard tiger tarsus was obtained through MRI. The SE T1-weighted images showed the best evaluation of the cortical, subchondral and trabecular bone of the tibia, fibula, tarsus and metatarsus bones. Nonetheless, the GE STIR T2-weighted images allowed us to better visualize the articular cartilage and synovial fluid. In both MRI pulse sequences, the ligaments and tendons appeared with low signal intensity compared with muscles that were visible with intermediate signal intensity.

**Conclusions:**

The results of this CT and MRI study of the Bengal tiger tarsal joint provide some valuable anatomical information and may be useful for diagnosing disorders in this large non-domestic cat.

## Background

There are two diagnostic techniques that can be regarded as some of the most prominent ones in the field of morphological and clinical evaluation of the musculoskeletal system: computed tomography (CT) and magnetic resonance imaging (MRI). CT is a better option when the goal is to supply details of the osseous structures of the tarsal joint [1]. On the other hand, when it comes to observing soft tissues and fluids, MRI is specially suited for the task, as it also provides particularly good displays of tendons and tendon sheaths, ligaments, synovial membrane, cartilage and bone [2]. In veterinary medicine, both techniques have been used in anatomical studies of two mammal groups in the tarsal region (equines [2–6] and canines [7–9]). Also, the clinical application of these techniques (CT [10–12] and MRI [13–15]) could be proved during the assessment of musculoskeletal disorders related to these joints and associated structures of these two groups. In the case of felines, we can refer to CT having been used for transverse implant placement [16] and for the diagnosis of infections [17] in the tarsus. In tigers, previous reports detail the radiological findings in the diagnosis of fracture of the tibia and fibula [18] and anatomical assessment of the stifle joint using MRI [19], but there is no publications describing the results of CT and MRI anatomy of the tarsal joint in these species. The tarsal joint of these species is considerably complex, as it is constituted by the tibia, fibula, tarsal bones, metatarsal bones and the ligaments and fibrocartilage that maintain the bones attached together. Nevertheless, no thorough CT or MRI anatomic tarsal joint studies of non-domestic felines have been carried out so far, and that is why performing an adequate CT and MRI anatomical identification could prove extremely useful for the understanding of injuries depicted in felines [20–23]. This research has as principal objective the execution of a detailed anatomical description of a Bengal tiger’s tarsal joint by CT, MRI and anatomical dissections.

## Methods

### Animals

In order to carry out the present piece of research, the cadaver of a captive female 6-year old Bengal tiger (105 kg) that perished due to respiratory disease was referred from the Cocodrilos Park zoo (Canary Islands, Spain) to the Veterinary Faculty of Las Palmas de Gran Canaria University.

### Computed tomography

With the objective of carrying out the CT, a 16-slice helical CT scanner (Toshiba Astelion, Toshiba Medical System, Madrid, Spain) was employed. A standard clinical protocol (120 kVp, 80 mA, 512 X 512 matrix and 283 × 283 field of view) was used to obtain the images of the animal, which had been placed in right lateral recumbence. Transverse 3-mm thick images with an inter-slice space of 1.5 mm were obtained and transferred to a DICOM workstation. We applied bone and soft tissue window settings (WW 3000/WL 500 and WW 400/WL 60, respectively) to obtain the CT images. By means of a standard DICOM 3D format (OsiriX MD, Geneva, Switzerland), we were able to use the original data to generate three-dimensional surface reconstructed images of the right tarsus joint.

### Magnetic resonance imaging

A magnet operating a field of 0.2-Tesla (Vet-MR Esaote, Genova, Italy) was used to carry out the MRI. The tiger was also positioned in right lateral recumbence, and the right tarsus joint was examined via MRI. In this study, SE T1-weighted and GE STIR T2-weighted pulse sequences were selected to generate transverse, sagittal and dorsal MRI planes. The basic technical parameters of the MRI study are displayed in Table 1.

**Table 1:** Basic technical parameters used in this MRI study

	SE T1	GE T2 STIR
Echo time (ms)	Transverse plane: 26	Transverse plane: 25
	Sagittal plane: 26	Sagittal plane: 25
	Dorsal plane: 26	Dorsal plane: 25
Repetition time (ms)	Transverse plane: 800	Transverse plane: 1680
	Sagittal plane: 800	Sagittal plane: 1680
	Dorsal plane: 800	Dorsal plane: 1680
Slice thickness (mm)	Transverse plane: 6	Transverse plane: 6
	Sagittal plane: 5	Sagittal plane: 5
	Dorsal plane: 4.5	Dorsal plane: 4.5
Interslice spacing (mm)	Transverse plane: 6.5	Transverse plane: 6.5
	Sagittal plane: 5.5	Sagittal plane: 5.5
	Dorsal plane: 5	Dorsal plane: 5
Acquisiton matrix	Transverse plane: 288/173	Transverse plane: 288/173
	Sagittal plane: 288/173	Sagittal plane: 288/173
	Dorsal plane: 288/173	Dorsal plane: 288/173

### Anatomic evaluation

Gross anatomical dissections of the right hind limb and its tarsal joint were performed after the performance of the imaging procedures, in order to facilitate the anatomical structures identification tasks and to establish comparisons with the CT and MRI images. We also resorted to veterinary anatomy manuals [24, 25] and to Bengal tarsal bones. Finally, in order to conform to the anatomical nomenclature [26], we labelled the different tarsal joint structures.

## Results

### Gross anatomical dissections

**Fig. 1:**
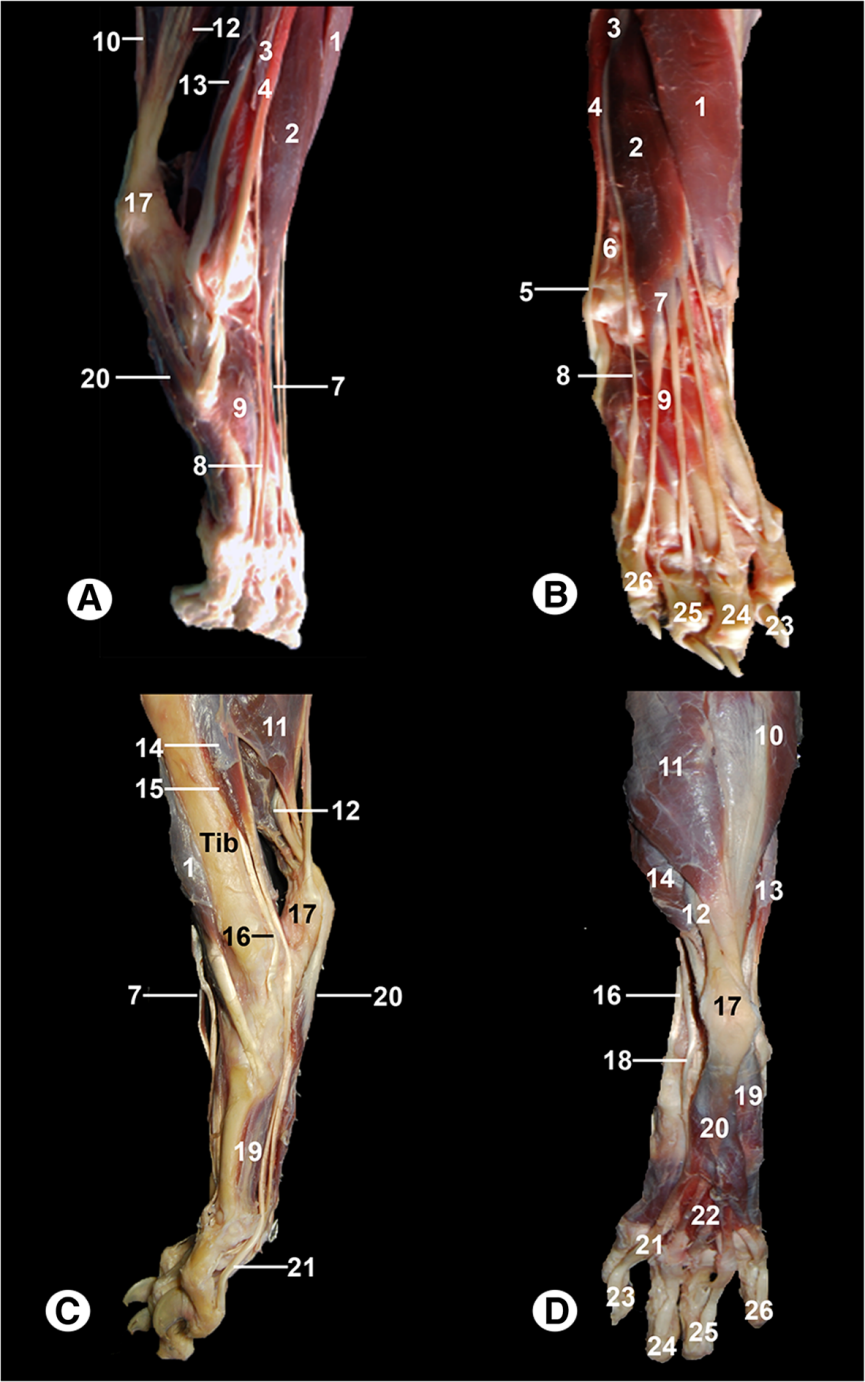
Gross anatomical dissections of the Bengal tiger right hind limb: **a** lateral aspect, (**b**) dorsal aspect, (**c**) medial aspect and (**d**) plantar aspect. 1 = cranial tibial muscle, 2 = long digital extensor muscle, 3 = lateral digital extensor muscle, 4 = long fibular muscle, 5 = long fibular tendon, 6 = short fibular muscle, 7 = long digital extensor tendon, 8 = lateral digital extensor tendon, 9 = short digital extensor muscle, 10 = gastrocnemius muscle lateral head, 11. gastrocnemius muscle medial head, 12 = superficial digital flexor muscle, 13 = lateral digital flexor muscle, 14 = medial digital flexor muscle, 15 = caudal tibial muscle, 16 = caudal tibial tendon, 17 = common calcaneal tendon, 18 = medial digital flexor muscle, 19 = interosseous muscles, 20 = superficial digital flexor tendon, 21 = deep digital flexor tendon, 22 = lumbrical muscles, 23 = 2nd digit, 24 = 3rd digit, 25 = 4th digit, and 26 = 5th digit

**Fig. 2:**
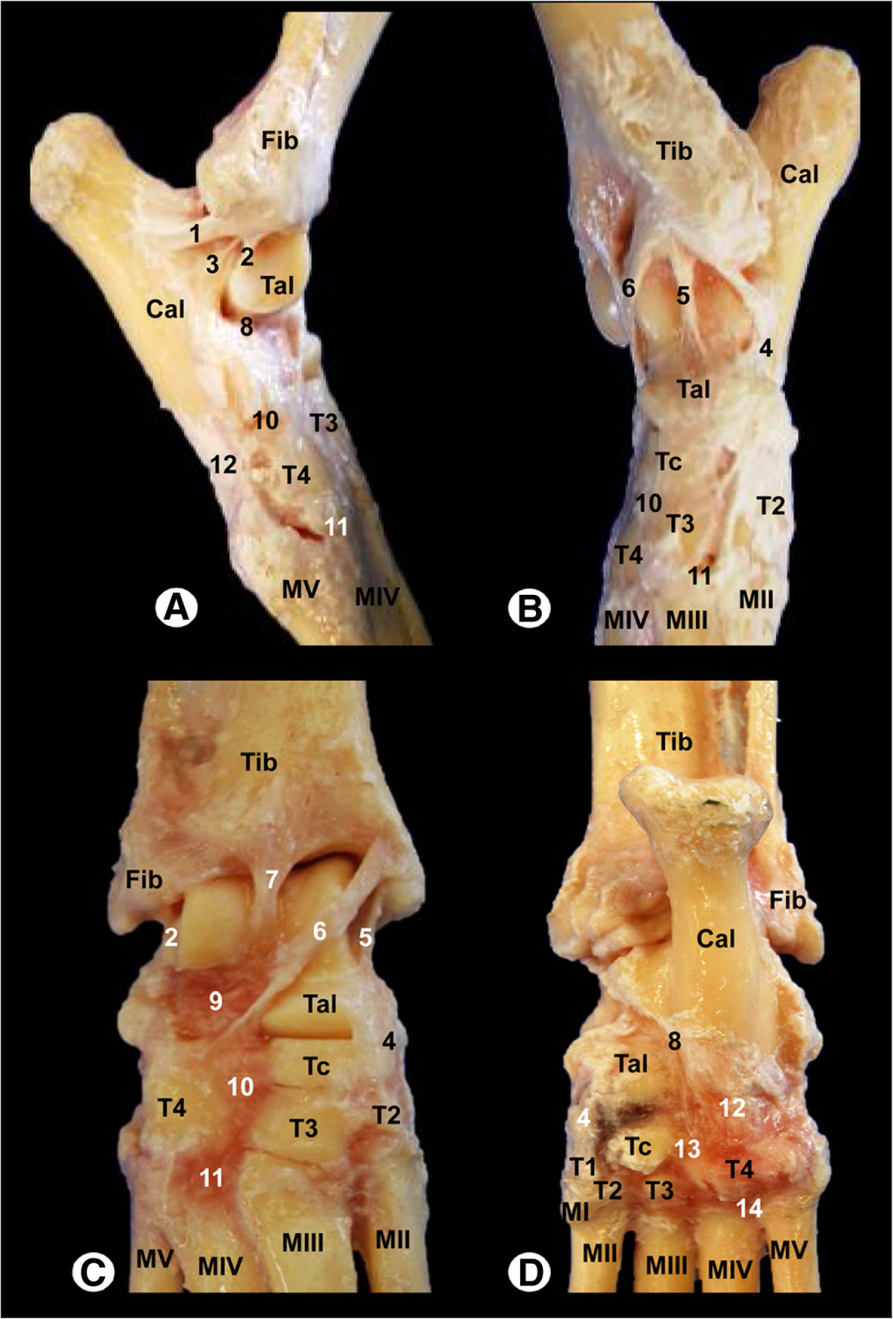
Gross anatomical dissections of the Bengal tiger right tarsal joint: **a** lateral aspect, (**b**) medial aspect, (**c**) dorsal aspect and (**d**) plantar aspect. Tib = tibia, Fib = fibula, Cal = calcaneus, Tal = talus, Tc = central tarsal bone, T1 = 1st tarsal bone, T2 = 2nd tarsal bone, T3 = 3rd tarsal bone, T4 = 4th tarsal bone, MI = 1st metatarsal bone, MII = 2nd metatarsal bone, MIII = 3rd metatarsal bone, MIV = 4th metatarsal bone, MV = 5th metatarsal bone. 1 = long lateral collateral ligament, 2 = short lateral collateral ligament (talofibular part), 3 = short lateral collateral ligament (calcaneofibular part), 4 = long medial collateral ligament, 5 = short medial collateral ligament (tibiotalar part), 6 = short medial collateral ligament (tibiocentral part), 7 = dorsal extensor retinaculum, 8 = talocalcaneal interosseous ligament, 9 = talocalcaneocentral ligament, 10 = dorsal intertarsal ligament, 11 = dorsal tarsometatarsal ligament, 12 = long plantar ligament, 13 = plantar intertarsal ligament, and 14 = plantar tarsometatarsal ligament

Gross anatomical dissections from different aspects of the right hind limb (Fig. 1) and right tarsal joint (Fig. 2) are presented. Several muscles and tendons are identified in Fig. 1. Thus, the gastrocnemius comprises a lateral and medial head. The origin of the lateral head of the gastrocnemius is the lateral supracondylar tuberosity of the femur and lateral sesamoid bone. The medial head originates from the medial supracondylar tuberosity of the femur and medial sesamoid bone. Their tendons insert into the calcaneal tuberosity, forming the common calcaneal tendon (1A, 1C and 1D), together with the superficial digital flexor muscle and the common tendons of the femoral biceps, gracilis, and semitendinosus muscles. The cranial tibial muscle was identified as extending from the lateral tibial condyle and tibial tuberosity and terminating medially on the base of the 2nd metatarsal and 1st tarsal bones (Fig. 1a, b and c). The long fibular muscle was visible, and it arose on the lateral collateral ligament of the femorotibial joint, lateral tibial condyle and fibular head and terminated on the base of each metatarsal bone (Fig. 1a and b). The short fibular muscle was found to extend from the lateral border of the tibia and the distal portion of the fibula to the dorsolateral surface at the base of the 5th metatarsal bone (Fig. 1b). The long digital extensor muscle arose from the femoral extensor fossa and the tendon split to terminate on the distal phalanx of each digit (Fig. 1a, b and c). The lateral digital extensor muscle was visible from the proximal portion of the fibula, and it terminated on the 5th digit (Fig. 1a and b). The short digital extensor muscle arose from the dorsal tarsal ligaments and calcaneus. Its tendons terminated by joining the long digital extensor tendons, which terminated on the 2nd to 5th digits (illustrated in Fig. 1a and b). The superficial digital flexor muscle arose at the supracondylar femoral fossa. It passes enclosed between the two heads of the gastrocnemius and its tendon forms a cap, which is attached to the calcaneal tuberosity by a medial and lateral retinaculum, and the tendon continued distally, dividing into four digital branches that terminated in the middle phalanx of the 2nd to 5th digits (Fig. 1a, c and d). The lateral digital flexor muscle and the medial digital flexor muscle compose the deep digital flexor muscle. The lateral digital flexor muscle arose from the caudal surface of the fibula, interosseous membrane of the leg and adjacent part of the tibia. The medial digital flexor muscle arose from the head of the fibula and popliteal line of the tibia. Its tendons formed the deep digital flexor tendons and terminated on the flexor surface of the distal phalanx of each digit (Fig. 1a, c and d). The caudal tibial muscle was visible from the fibular head, and it terminated on the medial collateral ligament (Fig. 1c). The interosseous muscles arose from the proximal portion and plantar surface of the 2nd to 5th metatarsal bones and terminated on the proximal sesamoid bones (Fig. 1c and d). The lumbrical muscles were visible at the level of the metatarsal bones between the deep digital flexor tendons (Fig. 1d).

In the gross dissections of the right tarsal joint, the tibia, the fibula, the tarsal and the metatarsal bones were identified (Fig. 2a-d). The main stabilizing articular structures of the tarsocrural joint (between the tibia, fibula, talus and calcaneus bones) included the long lateral collateral ligament, which extended from the lateral malleolus of the fibula to the calcaneus (Fig. 2a); the short lateral collateral ligament that connects the fibula and talus (talofibular part); and the fibula and calcaneus (calcaneofibular part), as illustrated in Fig. 2a-b. In addition, the long medial collateral ligament that connected the medial tibial malleolus to the 2nd tarsal bone, as well as the short medial collateral ligament, which extended from the medial tibial malleolus to the talus (tibiotalar part) and from the medial tibial malleolus to the central tarsal bone (tibiocentral part), are shown in the Fig. 2b-c. The main ligaments of the intertarsal joints were also visible. Thus, the talocalcaneal joint between the talus and calcaneus was observed (Fig. 2a and d). In addition, the talocalcaneocentral joint between the base of the talus, calcaneus and central tarsal bone and the centrodistal joint between the central tarsal bone and the distal tarsal bones were identified (Fig. 2b and c). Therefore, the calcaneoquartal joint is identified between the calcaneus and the 4th tarsal bone (Fig. 2a and d). Dorsal ligaments of the tarsus and metatarsus, such as the dorsal intertarsal and dorsal tarsometatarsal ligaments, were observed (Fig. 2a, b and c). The plantar intertarsal and tarsometatarsal ligaments were also identified (Fig. 2d), and the long plantar ligament that connects the plantar surface of the calcaneus, 4th tarsal bone and metatarsal bones was especially visible in Fig. 2a and d.

### Computed tomography

**Fig. 3:**
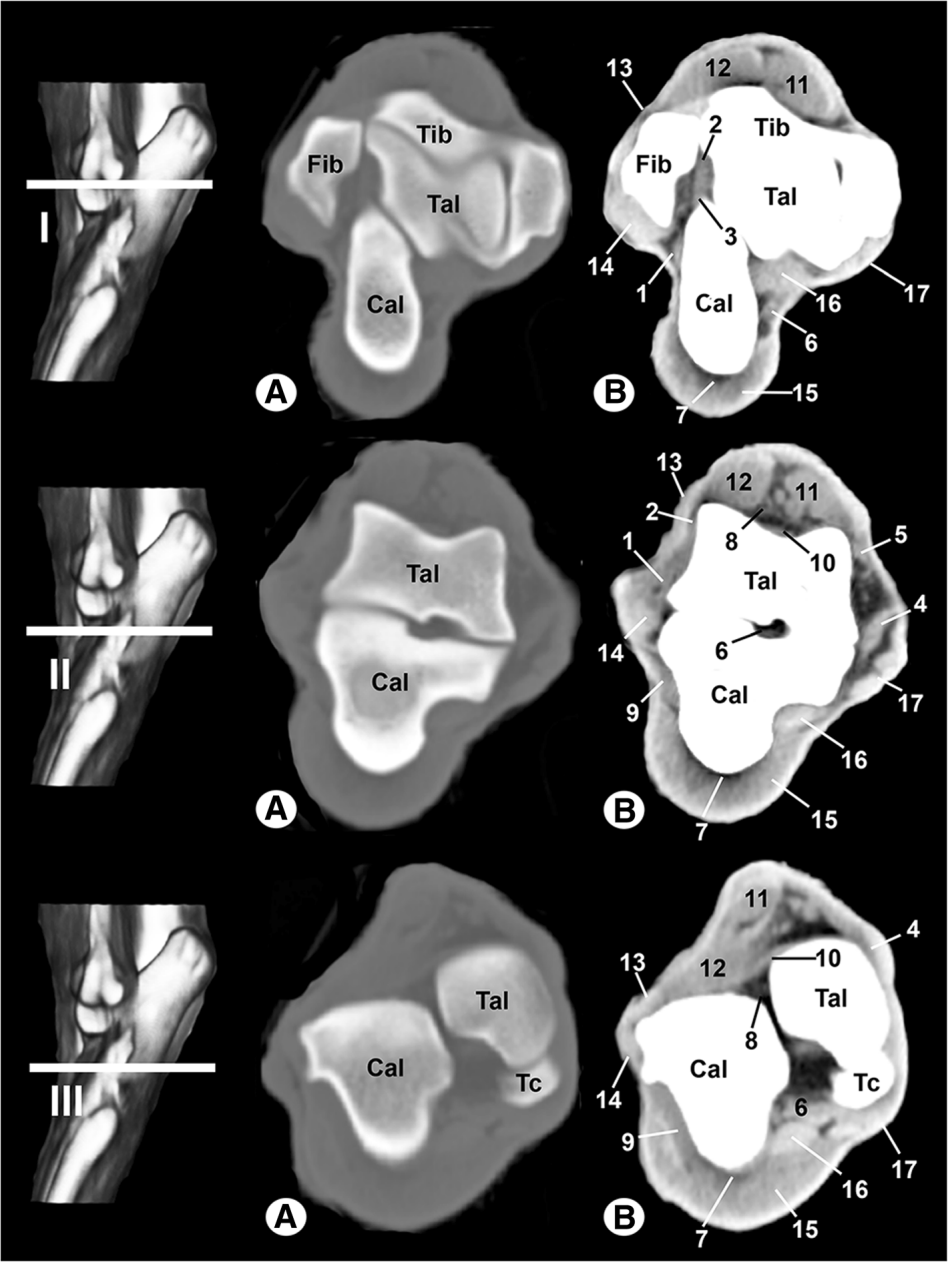
Transverse CT images of the right tarsal joint: **a** bone window and (**b**) soft-tissue window. Line depicts the section at the level of the tarsocrural joint (I), proximal third of the talocalcaneal joint (II) and distal third of the talocalcaneal joint (III). Tib = tibia, Fib = fibula, Tal = talus, Cal = calcaneus, Tc: central tarsal bone. 1 = long lateral collateral ligament, 2 = short lateral collateral ligament (talofibular part), 3 = short lateral collateral ligament (calcaneofibular part), 4 = long medial collateral ligament, 5 = short medial collateral ligament (tibiotalar part), 6 = talocalcaneal interosseous ligament, 7 = long plantar ligament, 8 = dorsal extensor retinaculum, 9 = calcaneoquartal ligament, 10 = talocalcaneocentral ligament, 11 = cranial tibial muscle, 12 = long digital extensor muscle, 13 = lateral digital extensor muscle, 14 = long fibular muscle, 15 = superficial digital flexor muscle, 16 = lateral digital flexor muscle, and 17 = medial digital flexor muscle

**Fig. 4:**
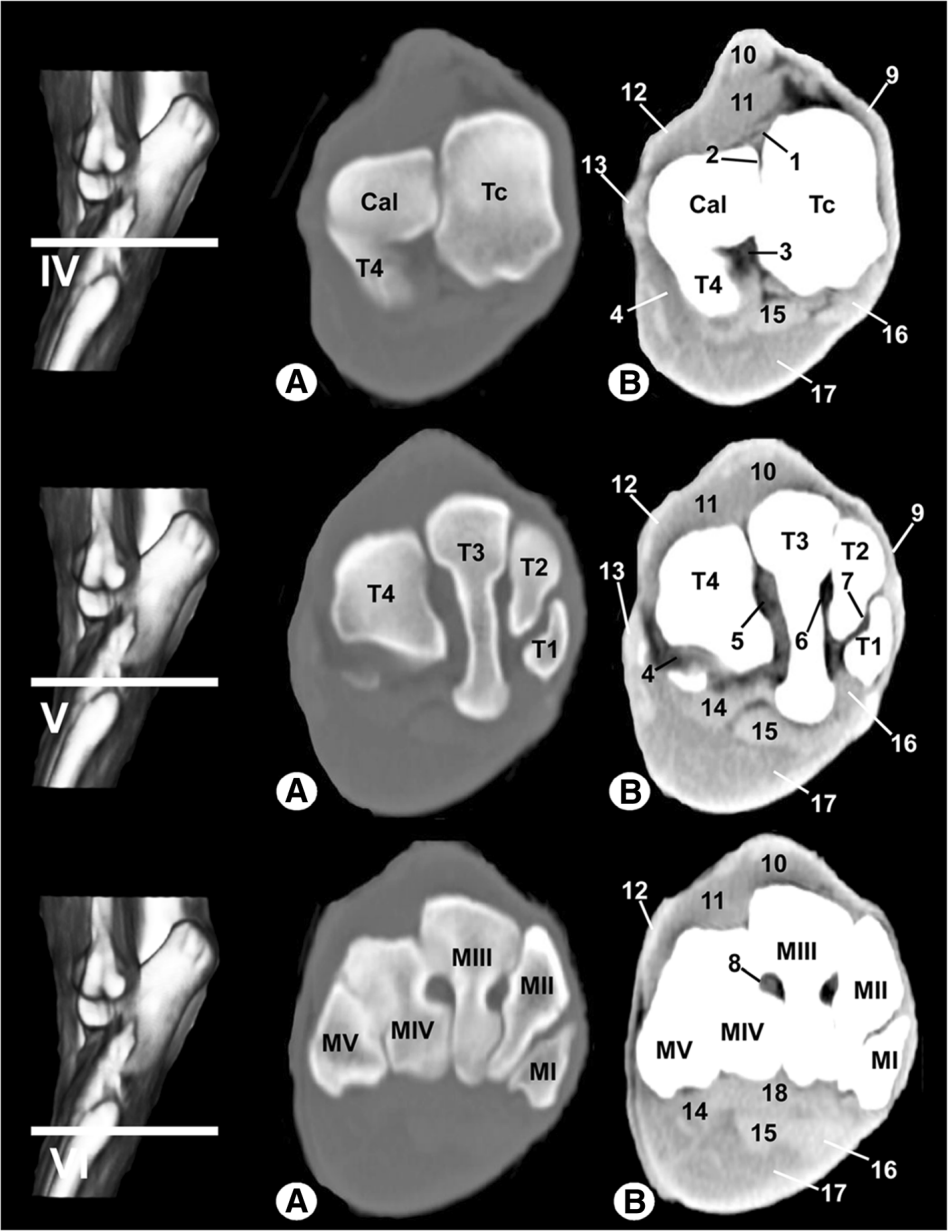
Transverse CT images of the right tarsal joint: **a** bone window and (**b**) soft-tissue window. Line depicts the section at the level of the talocalcaneocentral joint (IV), distal tarsal bones (V) and tarsometatarsal joint (VI). Cal = calcaneus, Tc = central tarsal bone, T1 = 1st tarsal bone, T2 = 2nd tarsal bone, T3 = 3rd tarsal bone, T4 = 4th tarsal bone, MI = 1st metatarsal bone, MII = 2nd metatarsal bone, MIII = 3rd metatarsal bone, MIV = 4th metatarsal bone, MV = 5th metatarsal bone. 1 = dorsal extensor retinaculum, 2 = interosseous intertarsal ligament between the calcaneus and central tarsal bone, 3 = long plantar ligament, 4 = calcaneoquartal ligament, 5 = interosseous intertarsal ligament between the T3 and T4, 6 = interosseous intertarsal ligament between the T2 and T3, 7 = interosseous intertarsal ligament between the T1 and T2, 8 = interosseous metatarsal ligament, 9 = cranial tibial tendon, 10 = long digital extensor tendon, 11 = short digital extensor muscle, 12 = lateral digital extensor tendon, 13 = long fibular tendon, 14 = tarsal fibrocartilage, 15 = lateral digital flexor tendon, 16 = medial digital flexor tendon, 17 = superficial digital flexor muscle, and 18 = interosseous muscle

**Fig. 5:**
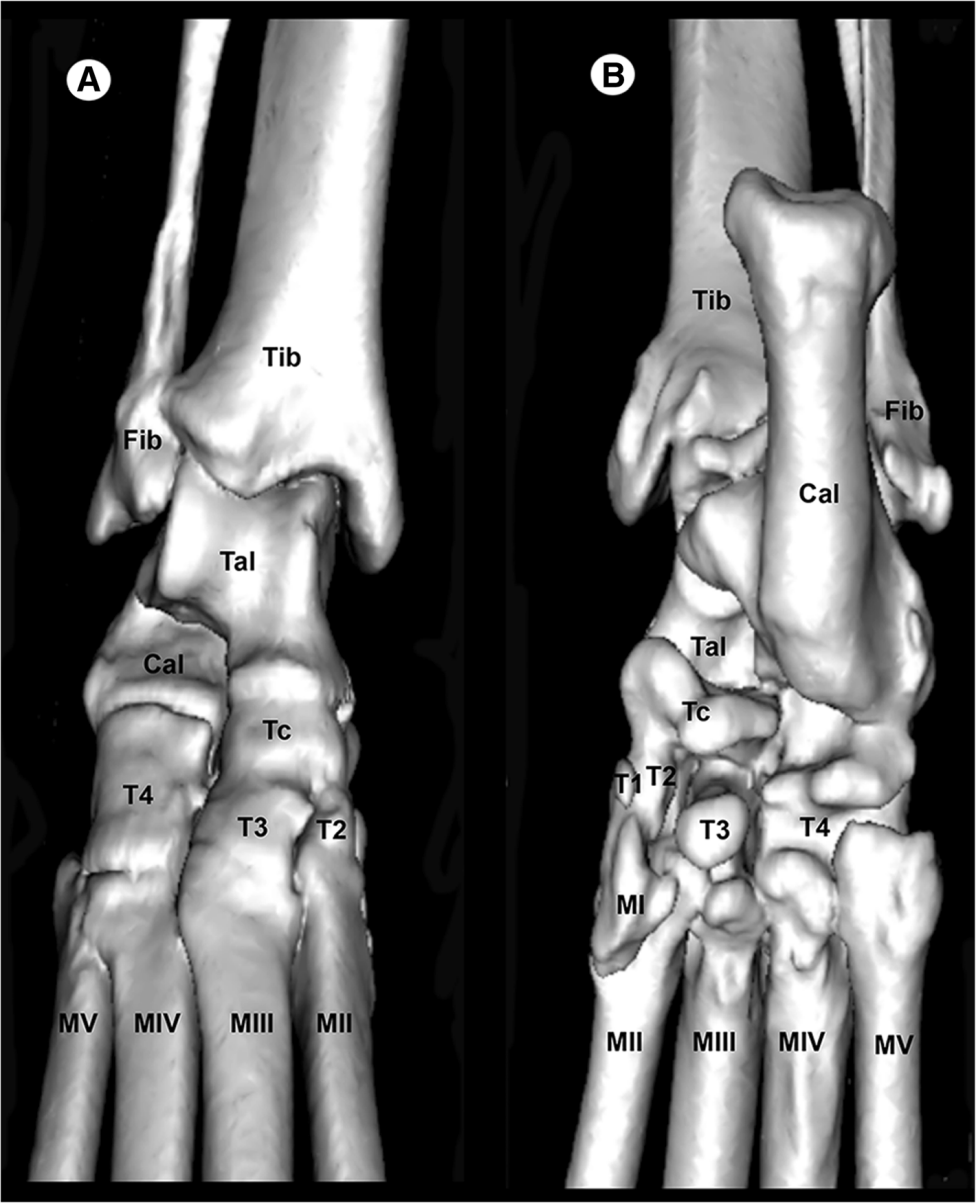
Three-dimensional surface reconstruction CT images of the right tarsal joint: **a** dorsal aspect and (**b**) plantar aspect. Tib = tibia, Fib = fibula, Cal = calcaneus, Tal = talus, Tc = central tarsal bone, T1 = 1st tarsal bone, T2 = 2nd tarsal bone, T3 = 3rd tarsal bone, T4 = 4th tarsal bone, MI = 1st metatarsal bone, MII = 2nd metatarsal bone, MIII = 3rd metatarsal bone, MIV = 4th metatarsal bone, and MV = 5th metatarsal bone

The CT images are shown in Figs. 3, 4, 5. In Figs. 3 and 4, transverse images are presented in a proximal to distal progression, from the tarsocrural joint (level I) to the tarsometatarsal joint (level VI). Three-dimensional surface reconstructions of the right tarsus joint are shown in Fig. 5. The CT images provided differentiation between the bones and the soft tissues in the tarsal joint. With bone window settings, the cortical and bone marrow of the tibia, fibula, tarsal and metatarsal bones were shown, and the trabecular bone was delineated (Figs. 3a and 4a). By contrast, the use of a soft tissue window differentiated the main soft tissue structures in the tarsal joint, such as the ligaments, muscles and tendons, which appeared with variable density, and the synovial fluid had a low attenuation (Figs. 3b and 4b). However, the osseous structures appeared with high attenuation and differentiation of the cortical bone from the bone marrow was not possible. On the three-dimensional surface reconstruction, all bones were identified by their high attenuation (Fig. 5).

### Magnetic resonance imaging

**Fig. 6:**
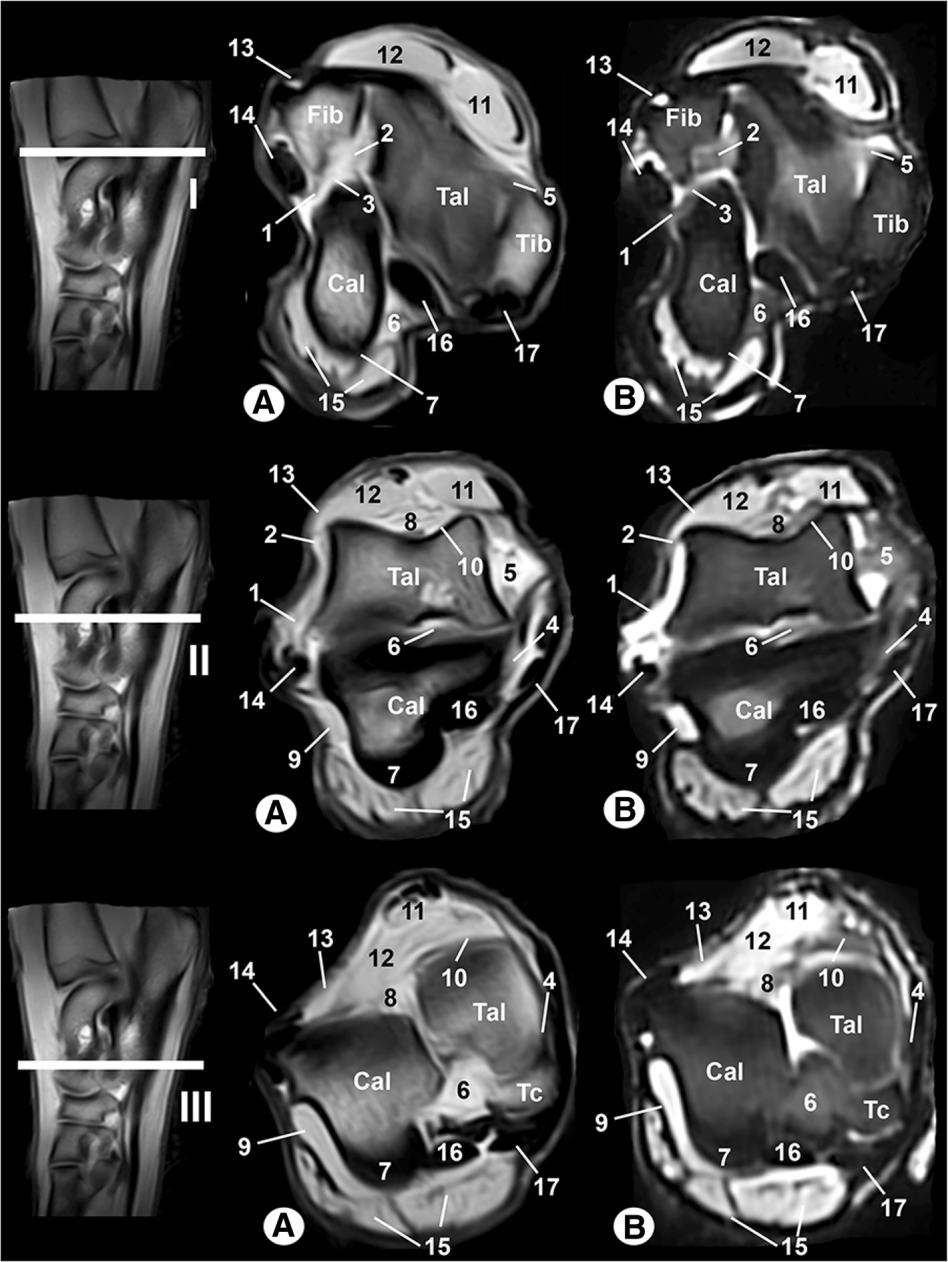
Transverse MR images of the right tarsal joint: **a** SE T1-weighted and (**b**) GE STIR T2-weighted. Line depicts the section at the level of the tarsocrural joint (I), proximal third of talocalcaneal joint (II) and distal third of talocalcaneal joint (III). Tib = tibia, Fib = fibula, Tal = talus, Cal = calcaneus, Tc = central tarsal bone. 1 = long lateral collateral ligament, 2 = short lateral collateral ligament (talofibular part), 3 = short lateral collateral ligament (calcaneofibular part), 4 = long medial collateral ligament, 5 = short medial collateral ligament (tibiotalar part), 6 = talocalcaneal interosseous ligament, 7 = long plantar ligament, 8 = dorsal extensor retinaculum, 9 = calcaneoquartal ligament, 10 = talocalcaneocentral ligament, 11 = cranial tibial muscle, 12 = long digital extensor muscle, 13 = lateral digital extensor muscle, 14 = long fibular muscle, 15 = superficial digital flexor tendon, 16 = lateral digital flexor muscle, and 17 = medial digital flexor muscle

**Fig. 7:**
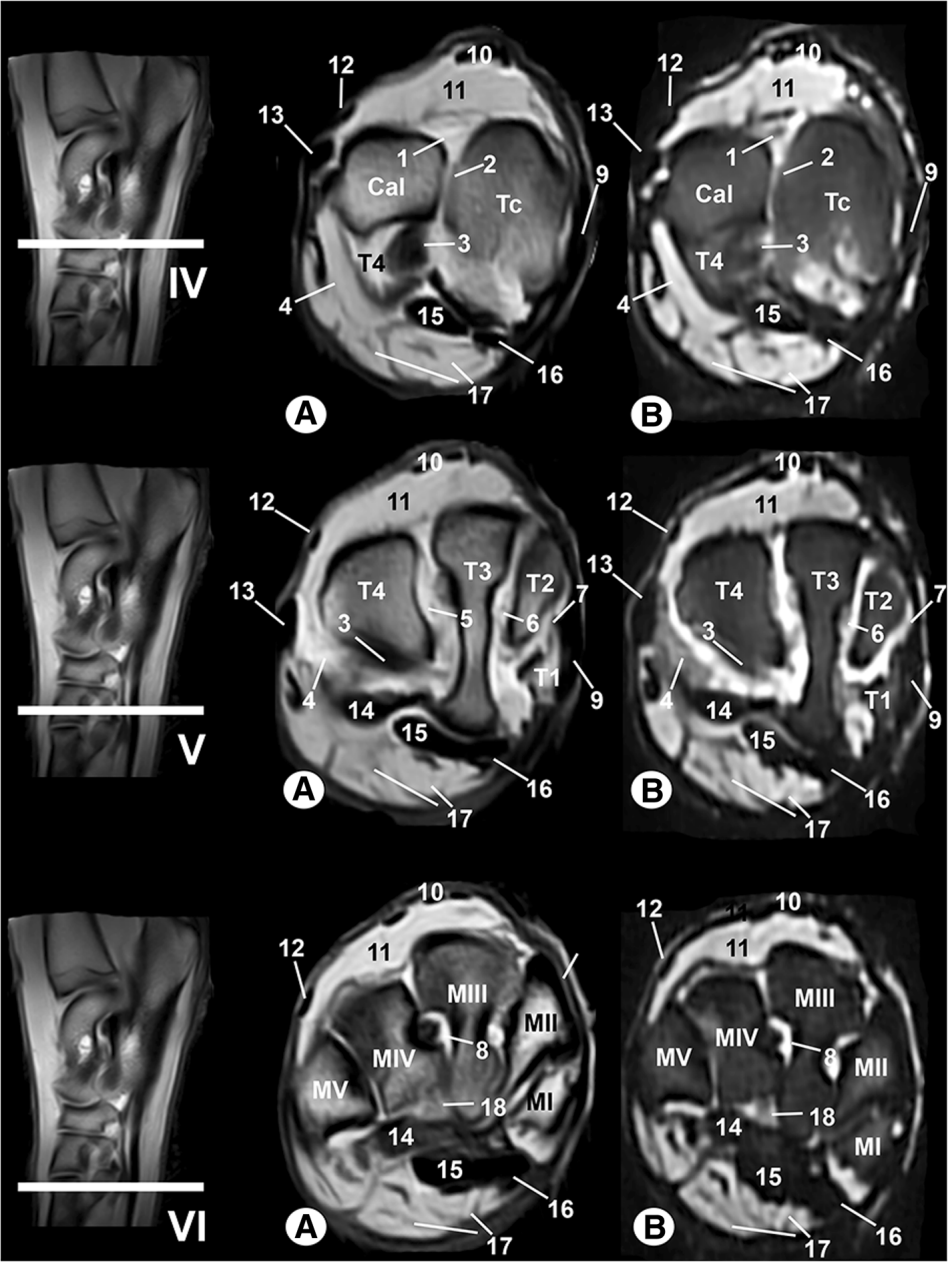
Transverse MR images of the right tarsal joint: **a** SE T1-weighted and (**b**) GE STIR T2-weighted. Line depicts the section at the level of the proximal tarsal bones (IV), distal tarsal bones (V) and metatarsal bones (VI). Cal = calcaneus, Tc = central tarsal bone, T1 = 1st tarsal bone, T2 = 2nd tarsal bone, T3 = 3rd tarsal bone, T4 = 4th tarsal bone, MI = 1st metatarsal bone; MII = 2nd metatarsal bone, MIII = 3rd metatarsal bone, MIV = 4th metatarsal bone, MV = 5th metatarsal bone. 1 = dorsal extensor retinaculum, 2 = interosseous intertarsal ligament between the calcaneus and central tarsal bone, 3 = long plantar ligament, 4 = calcaneoquartal ligament, 5 = interosseous intertarsal ligament between the T3 and T4, 6 = interosseous intertarsal ligament between the T2 and T3, 7 = interosseous intertarsal ligament between the T1 and T2, 8 = tarsometatarsal ligament, 9 = cranial tibial tendon; 10 = long digital extensor tendon, 11 = short digital extensor muscles, 12 = lateral digital extensor tendon, 13 = long fibular tendon, 14 = tarsal fibrocartilage, 15 = lateral digital flexor tendon, 16 = medial digital flexor tendon, 17 = superficial digital flexor tendon, and 18 = interosseous muscle

**Fig. 8:**
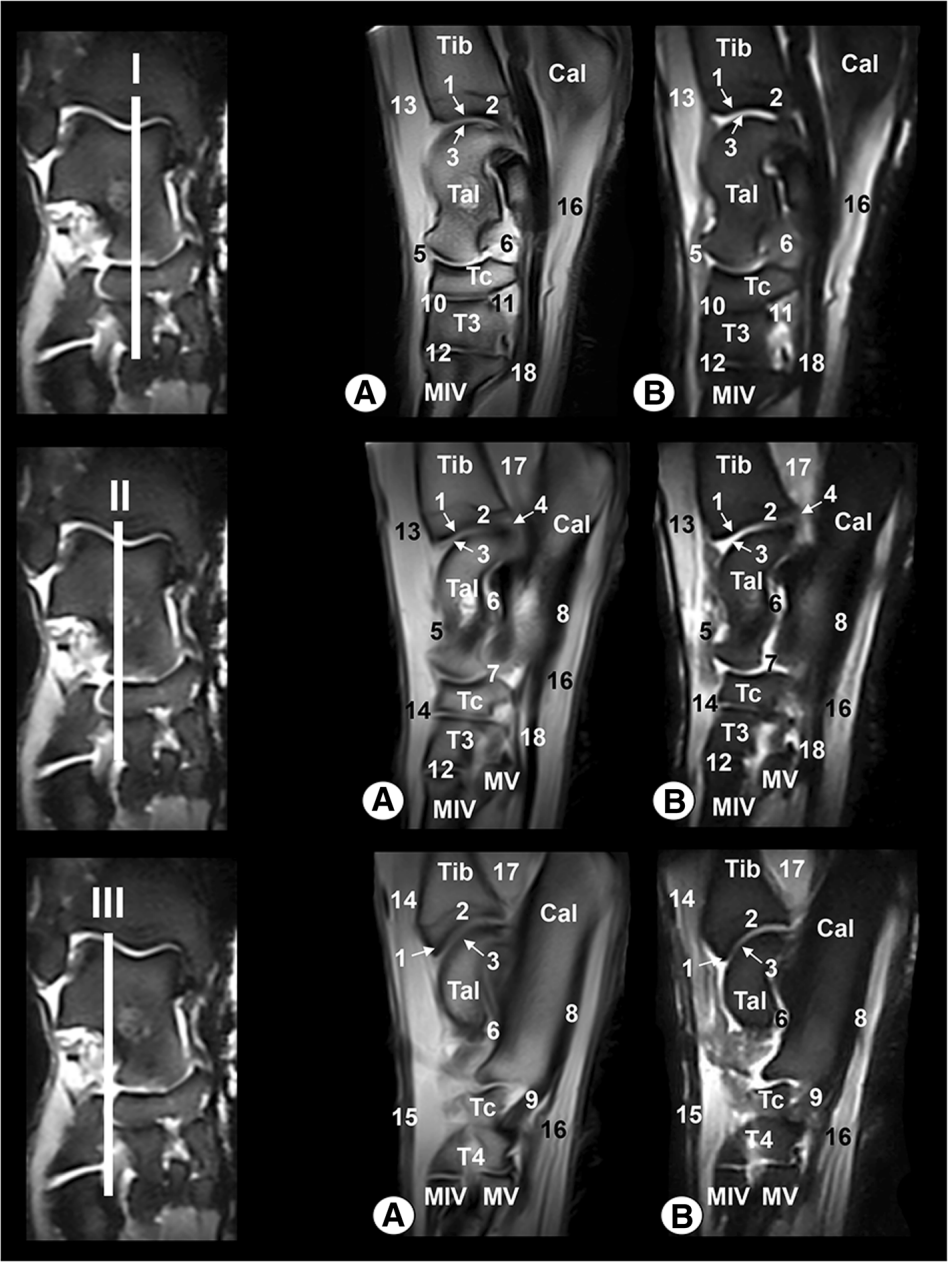
Sagittal MR images of the right tarsal joint: **a** SE T1-weighted and (**b**) GE STIR T2-weighted. Line depicts the section at the level of the medial third of talus (I), middle third of talus (II) and lateral third of talus (III). Tib = tibia, Tal = talus, Cal = calcaneus. Tc = central tarsal bone, T3 = 3rd tarsal bone, T4 = 4th tarsal bone, MIV = 4th metatarsal bone, MV = 5th metatarsal bone. 1 = subchondral bone, 2 = trabecular bone, 3 = articular cartilage, 4 = articular capsule, 5 = short medial collateral ligament (tibiocentral part), 6 = talocalcaneal interosseous ligament, 7 = talocalcaneocentral ligament, 8 = long plantar ligament, 9 = calcaneoquartal ligament, 10 = dorsal intertarsal ligament, 11 = plantar intertarsal ligament, 12 = dorsal tarsometatarsal ligament, 13 = cranial tibial muscle, 14 = long digital extensor muscle, 15 = long digital extensor tendon, 16 = superficial digital flexor muscle, 17 = deep digital flexor muscle, and 18 = deep digital flexor tendon

**Fig. 9:**
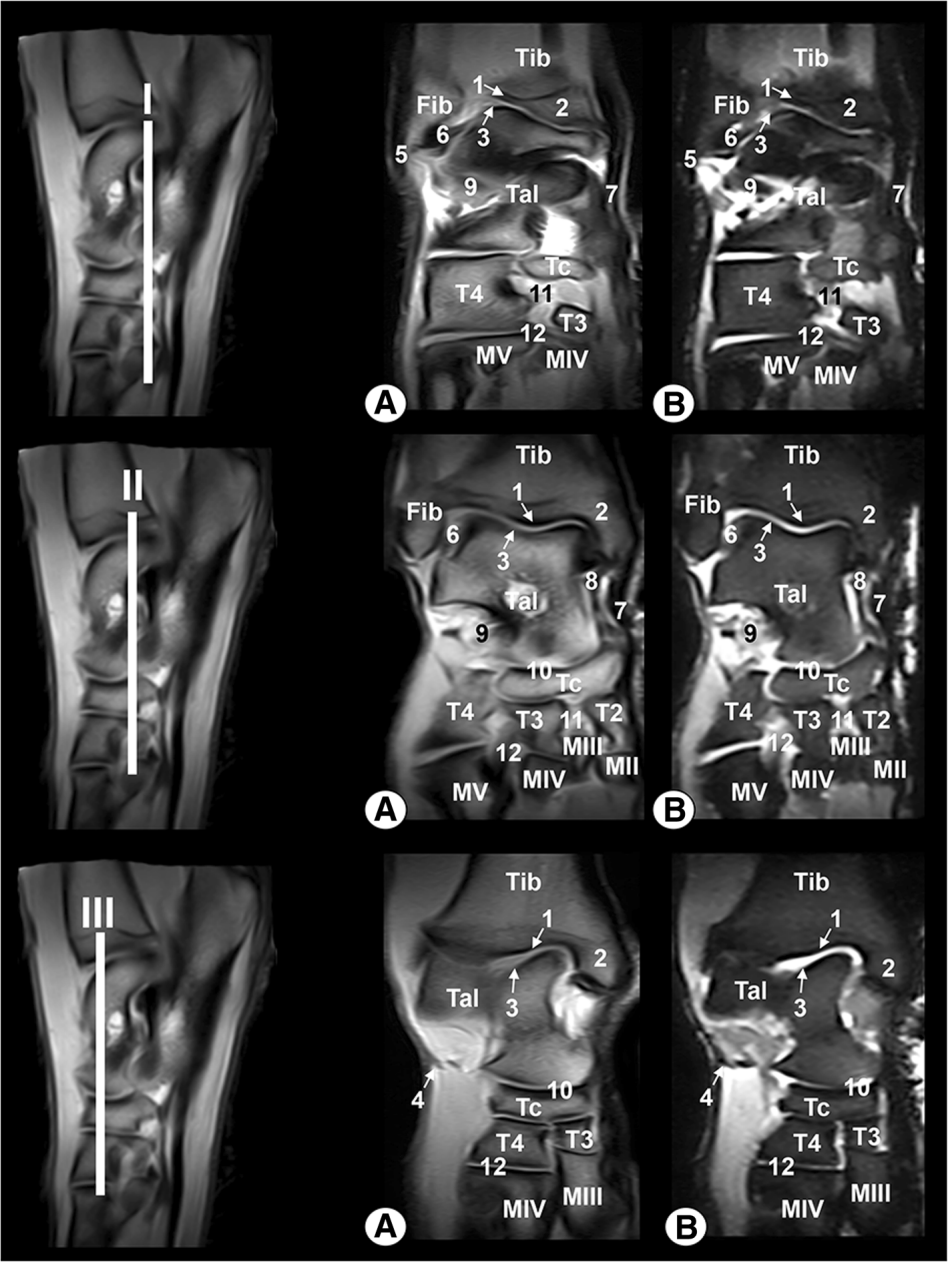
Dorsal MR images of the right tarsal joint: **a** SE T1-weighted and (**b**) GE STIR T2-weighted. Line depicts the level of section at the level of the plantar third of the central tarsal bone (I), plantar third of the talus (II) and dorsal third of the central tarsal bone (III). Tib = tibia, Fib = fibula, Tal = talus, Tc = central tarsal bone, T2 = 2nd tarsal bone, T3 = 3rd tarsal bone, T4 = 4th tarsal bone, MII = 2nd metatarsal bone, MIII = 3rd metatarsal bone, MIV = 4th metatarsal bone, MV = 5th metatarsal bone. 1 = subchondral bone, 2 = trabecular bone, 3 = articular cartilage, 4 = articular capsule, 5 = long lateral collateral ligament, 6 = short lateral collateral ligament (talofibular part), 7 = long medial collateral ligament, 8 = short medial collateral ligament (tibiotalar part), 9 = talocalcaneal interosseous ligament, 10 = talocalcaneocentral ligament, 11 = plantar intertarsal ligament, and 12 = plantar tarsometatarsal ligament

Clinically osseous and soft-tissue structures of the tarsus joint were identified and labelled in the four figures corresponding to the MR images (Figs. 6, 7, 8, 9). The transverse MR images are shown in Figs. 6 and 7. These figures are shown from the tarsocrural joint (level I) to the tarsometatarsal joint (level VI). In Fig. 8, the sagittal MR images are visible from the medial third of the talus (level I) to the lateral third of the talus (level III). Figure 9 is a composite of three images corresponding to the dorsal MR images beginning at the level of the plantar third of the central tarsal bone (level I) to the dorsal third of the central tarsal bone (level III). Low-field MRI provided good anatomical detail of the structures of the tarsus joint. On the SE T1-weighted images (Figs. 6a, 7, 8, 9a), the cortical and subchondral bone of the tibia, fibula, tarsal and metatarsal bones had a low signal intensity compared with the trabecular bone, which had high signal intensity. The articular cartilage and synovial fluid had intermediate signal intensity. On the GE STIR T2-weighted MR images (Figs. 6b, 7, 8, 9b), the cortical and subchondral bone appeared with negligible signal intensity. By contrast, the trabecular bone showed low signal intensity. The articular cartilage and the synovial fluid appeared with high signal intensity. A dark line corresponding to the subchondral bone allowed us to differentiate the signal intensity between the articular cartilage and the trabecular bone in both sequences, and this line was especially visible in the sagittal and dorsal MRI anatomical planes (Figs. 8 and 9).

In the MR images, several ligaments of the tarsal joint were also observed. Thus, the long and short lateral and medial collateral ligaments, as well as the talocalcaneal interosseous ligament of the tarsocrural joint, appeared with low signal intensity in the transverse (Figs. 6 and 7), sagittal (Fig. 8) and dorsal (Fig. 9) images. However, the dorsal plane provided the best views of the tarsal collateral ligaments, which appeared as linear, low-signal-intensity bands. The talocalcaneocentral and the calcaneoquartal ligaments were also observed, especially in the transverse (Figs. 6 and 7) and sagittal (Fig. 8) images. The centrodistal, intertarsal and tarsometatarsal ligaments had low signal intensity and were more clearly observed in the sagittal (Fig. 8) and dorsal (Fig. 9) images compared with the transverse plane (Figs. 6 and 7). However, in all anatomical MRI planes, due to the presence of synovial fluid or fat, some ligaments appeared with high or intermediate signal intensity (Figs. 6, 7, 8, 9). In both MRI sequences, the articular capsule appeared with low signal intensity and was visible especially in the sagittal (Fig. 8) and dorsal (Fig. 9) images.

In addition, several muscles, including the cranial tibial, long digital extensor, lateral digital extensor, long fibular, short fibular, short digital extensor, superficial digital flexor, deep lateral digital flexor, deep medial digital flexor and interosseous, were well defined and appeared with variable intermediate intensity in both sequences. However, the tendons appeared with dark grey to black signal intensities in the SE T1-weighted images and with a dark grey signal in the GE STIR T2-weighted images. These muscular structures were easily observed, especially in the transverse (Figs. 6 and 7) and sagittal (Fig. 8) planes compared with the dorsal images (Fig. 9).

## Discussion

First, we should refer to the fact that wildlife conservation has been taken as aim by several different scientific and academic disciplines, among which we should mention veterinary medicine. Their involvement in conservation dates back several decades, but the role of veterinarians has only attained remarkable recognition in the most recent times [27]. Now, going back to the tarsal joint of the Bengal tiger, it is important to make clear that it conforms a quite complex anatomical region (similar to that of other quadrupedal mammals), which hinders the task of performing physical examinations and clinical assessments of the anatomical structures of this particular area. In order to obtain images of this joint, radiography and ultrasonography have traditionally been used [28, 29]. Despite this, CT and MRI have progressively gained credit for their ability to provide more data to assess the osseous and soft tissue structures of the tarsal joint. In fact, when compared with radiography and ultrasonography, these techniques have proven to reliably provide images with good anatomic resolution, high contrast among different structures and an excellent tissue [2, 30].

For this research, a detailed algorithm which used a narrow window for soft tissue and a wide window for bone was utilized. The images allowed us to see the relationship between the medulla and the cortex thanks to the particular bone window settings that we had used. They also showed a perfectly delineated trabecular bone. In the case of the tarsal joint main soft tissue structures, they could be properly differentiated thanks to the soft tissue window. It is worth noting that these particular aspects have been previously described in camels [30], dogs [8] and horses [6]. The three-dimensional surface reconstructed images were also very helpful, as they allowed us to observe the different views and the most important surface features of the joint.

Now, with regard to the MRI, it was obtained via a low-field MRI magnet which provided us with an appropriate visualization of the normal structures of the tarsal joint. In the case of horses and dogs [4, 13–15], low-field intensity protocols for the study of the tarsal joint have been reported, as well as high-field magnet studies [2, 9]. Both have provided good resolution images. In our research, we used pulse sequences (SE T1-weighted and GE STIR T2-weighted) that made possible for us to adequately observe the main anatomical structures of several joints (tarsocrural, intertarsal and tarsometatarsal), most noticeably in the case of the SE T1-weighted images related to the GE STIR T2-weighted images. The tarsus joints of dogs [9] and horses [4, 5, 14] have been studied by using a similar MRI sequences, although Bolt et al. [15] only applied SE T1-weighted images to diagnose a central tarsal bone fracture in a horse.

The Bengal tiger tarsus joint observed in this study was imaged in three anatomical planes: transverse, sagittal and dorsal. These same planes have previously been used in veterinary medicine by different researchers [4, 9, 14, 15], although Latorre et al. [2] showed images only in the sagittal and transverse planes. In our research, the lateral and medial collateral ligaments of the tarsocrural joint showed better definition in the dorsal plane. The sagittal and dorsal planes provided the best views of the talocalcaneal interosseous ligament and the talocalcaneocentral ligament, whereas the centrodistal joint was observed especially in the dorsal MRI plane. In our case, the dorsal and sagittal planes provided very good views of the talocalcaneal interosseous and talocalcaneocentral ligaments, whereas the dorsal plane allowed us to better observe the plantar intertarsal ligaments and the centrodistal and tarsocrural joints (as well as the tarsocrural joint’s lateral and medial collateral ligaments). The sagittal MRI plane provided us a better view of other structures, such as the dorsal intertarsal and the long plantar and calcaneoquartal ligaments. In the case of the interosseous intertarsal ligaments, they were best delineated in the transverse MRI plane. In addition, the interosseous intertarsal ligaments were better delineated in the MRI transverse plane. These tarsal joint observations have also been reported in horse [4] and dog [9]. The major drawback of the use of this imaging technique is that the presence of synovial fluid dimmed the definition of articular cartilage tissue with similar signal intensity.

The identification of the ligaments, muscles, tendons and osseous structures of the Bengal tiger in the CT and MRI images presented in this research was facilitated by the conduction of gross anatomical dissections of its right hind limb and tarsus joint. These two imaging techniques are becoming increasingly available for use in veterinary medicine tasks regarding the musculoskeletal system, although the obtainment of images in endangered animals like the Bengal tiger is severely hindered by their hefty cost and limited availability [19]. Nevertheless, the small risk degree which its application entails might allow us to justify its use in these captive threatened species. Finally, we should add that this study has provided the first ever conducted anatomical description of the tarsus of a Bengal Tiger by means of helical CT and low-field MRI and that the establishment of CT and MRI protocols in live Bengal tigers could be highly beneficial to ensure a better assessment of the tarsal joint by using 1.5 mm CT helical slices and high-field MRI equipment.

## Conclusions

This study provides some useful anatomical information on the Bengal tiger tarsus joint. This study can serve as a basic anatomic reference aid to clinicians in the interpretation of injuries and the pathology of this joint in other animals of this species.

## Abbreviations

CT: Computed tomography
GE: Gradient-echo
MR: Magnetic resonance
MRI: Magnetic resonance imaging
SE: Spin-echo
TE: Echo time
TR: Repetition time
